# Intrarater Reliability and Analysis of Learning Effects in the Y Balance Test

**DOI:** 10.3390/mps6020041

**Published:** 2023-04-07

**Authors:** Olli Kattilakoski, Noora Kauranen, Mari Leppänen, Pekka Kannus, Kati Pasanen, Tommi Vasankari, Jari Parkkari

**Affiliations:** 1 Tampere Research Center of Sports Medicine, UKK Institute, 33500 Tampere, Finland; 2 Faculty of Medicine and Health Technology, Tampere University, 33014 Tampere, Finland; 3 Research Committee, Tampere University Hospital, 33521 Tampere, Finland; 4 Faculty of Kinesiology, Sport Injury Prevention Research Centre, University of Calgary, Calgary, AB T2N 1N4, Canada; 5 Alberta Children’s Hospital Research Institute, University of Calgary, Calgary, AB T2N 1N4, Canada; 6 McCaig Institute for Bone and Joint Health, University of Calgary, Calgary, AB T2N 1N4, Canada; 7 UKK Institute, 33500 Tampere, Finland; 8 Faculty of Sport and Health Sciences, University of Jyväskylä, 40014 Jyväskylä, Finland

**Keywords:** clinical balance tests, dynamic postural control, postural balance, reliability

## Abstract

While the general reliability of the Y balance test has been previously found to be excellent, earlier reviews highlighted a need for a more consistent methodology between studies. The purpose of this test–retest intrarater reliability study was to assess the intrarater reliability of the YBT using different methodologies regarding normalisation for leg length, number of repetitions, and score calculation. Sixteen healthy adult novice recreational runners aged 18–55 years, both women and men, were reviewed in a laboratory environment. Mean calculated scores, intraclass correlation coefficient, standard error of measurement, and minimal detectable change were calculated and analysed between different leg length normalisation and score calculation methods. The number of repetitions needed to reach a plateauing of results was analysed from the mean proportion of maximal reach per successful repetition. The intrarater reliability of the YBT was found to be good to excellent, and it was not affected by the method of score calculation or leg length measurement. The test results plateaued after the sixth successful repetition. Based on this study, it is suggested to use anterior superior iliac spine–medial malleolus length for leg length normalisation because this method was proposed in the original YBT protocol. At least seven successful repetitions should be performed to reach a result plateau. The average of the best three repetitions should be used to mitigate possible outliers and account for the learning effects seen in this study.

## 1. Introduction

Postural control has been proposed as a modifiable risk factor for lower extremity injuries [1,2]. While most available research on the matter has been able to identify different balance-related variables as risk factors for lower extremity injury [3,4,5], there have also been findings to the contrary [6]. The relative variability in results may be associated with how balance is tested. In past studies, there have been three dominating methods of testing balance—specifically, static or dynamic balance tests—using centre of pressure (COP) measures with differing force plate platforms, Biodex Balance System (Biodex Medical Systems, Shirley, NY), and different versions of the Star Excursion Balance Test (SEBT) [7,8,9,10,11]. It was suggested that there is no relationship between static and more dynamic balance tests [12].

The SEBT and its generally used modification, the Y balance test (YBT), are viewed as measurements of dynamic balance that require strength, flexibility, and proprioception [12,13]. The YBT consists of unilateral lower extremity reaching tasks performed in three out of eight original SEBT directions: the anterior, posteromedial, and posterolateral directions [14]. The maximal distance reached in every test direction is measured in centimetres and a composite score that is the mean result of all test directions can be calculated [13,15]. Reaching leg length can be used to normalise the result by dividing the absolute measurement by reaching leg limb length in centimetres multiplied by 100 [3,13]. The advantages of YBT compared with COP measurements are its ease of use and its availability in terms of low cost. While there are commercial ready-to-use kits available, the test can also be conducted with as few as three measurement tapes and a trained rater.

The general inter- and intrarater reliability of SEBT/YBT was found to be excellent in a systematic review including a total of nine studies on reliability [15]. However, in the practical implications, authors of the review highlighted the need for a consistent methodology including the use of leg length normalisation to be used in a clinical setting, if results are to be compared, as different methodological techniques may produce different values. The biggest variation in methodology was found in the number of both practise and collection repetitions, which ranged from zero to six and three to seven, respectively, in the included studies [15]. Other reported differences in methodology between studies were with regard to body position (six out of nine studies required hands to remain on hips, two studies required stance legs’ heel to remain flat on ground, and studies were split on stance leg placement) or use of normalisation by leg length (six out of nine studies) [15]. Another study identified two main ways to measure leg length for normalisation in earlier studies: anterior superior iliac spine (ASIS) to medial malleolus and ASIS to lateral malleolus [13]. Normalisation using total body height was also explored but correlation was found to be stronger for limb length [16].

Hence, there is a need for a reliability study comparing different methodological choices, especially regarding the number of repetitions, to give clinicians further guidance on the consistent execution of YBT to help make results more comparable. Therefore, the purpose of this YBT reliability study was to assess the most consistent methodology regarding normalisation for leg length, number of repetitions, and calculation of score measured by intrarater reliability.

## 2. Materials and Methods

### 2.1. Participants

This study was part of a larger randomised controlled trial investigating prevention of running-related injuries in novice recreational runners. The study population consisted of 16 healthy female and male adult volunteers with no previously identified issues with balance. Written informed consent was obtained from all participants. Participants were novice recreational runners recruited via announcements on the institute’s website and social media channels. The inclusion criteria were as follows:

1. Participants had to be adults aged 18 to 55 years;
2. They were required to be nonsmokers with no history of smoking or nicotine snus use in the previous 6 months;
3. They defined running as their primary way of exercising;
4. They had less than 2 years of experience running weekly with a limit of 20 kilometres or less per week;
5. They had no musculoskeletal injuries and reported no back or lower limb pain in the previous 3 months;
6. They reported no underlying diseases that could hinder running exercise and were required to be able to continuously run at least 3 kilometres or 20 min.

Participants reported weekly frequency of running ranging from 1 to 2 times, training session length ranging from 20 to 60 min, with distances ran ranging from 2.5 to 7 km per training session.

### 2.2. Test Procedure

Study participants were tested using a modified version of the YBT protocol originally proposed by Plisky et al. [13]. YBT was chosen as a test because it is a commonly used test to measure dynamic balance among athlete and recreational populations [4,17]. The following modifications to the original protocol were made: stance leg heel had to stay grounded, participants were instructed to keep hands on hips, starting leg and the order of reaching directions were randomised, tape measure was used instead of the proposed YBT kit, and the number of practise repetitions was limited to one per direction. The test was repeated 7 to 12 days after the first test. Before completing the balance test, the participants performed a warmup and a three-dimensional running gait analysis over ground. The warmup consisted of 5 min of walking followed by 5 min of jogging at a self-selected speed, and the running gait analysis included 20–30 min of short bursts of running.

Height (KaWe 44440 wall-mounted stadiometer, KIRCHNER & WILHELM GmbH + Co. KG, Asperg, BW, Germany), body mass (KERN MPB 300K100 personal floor scale, KERN & SOHN GmbH, Balingen-Frommern, BW, Germany) and leg length (plastic tape measure) were measured using the metric system before both the test and retest. Leg length was measured in two different ways as follows: from the anterior superior iliac spine (ASIS) to the tip of the hallux and from the ASIS to the medial malleolus. In the ASIS–hallux length measurement, the participant started by lying in the supine position with flexed knees and was then instructed to lift the pelvis and return to this starting position. ASIS-hallux length measurement was then recorded for one leg at a time by the rater passively extending the participant’s leg while instructing the participant to actively extend the ankle. The ASIS–medial malleolus length was measured with the participant standing with their back against a wall with a stance of feet 9 cm apart. The dominant leg was determined using three tasks: First, the participant was asked to kick a ball; then, they were asked to rise to a stair; and, finally, they were gently nudged forward if needed. The leading leg was registered, and the leading leg of at least two out of three tasks was registered as the dominant one.

Two raters working in cooperation measured and recorded the results from participants at both testing times. One rater supervised the performance and fulfilment of conditions for a successful repetition while the other took the measures. The length of the reach was measured by observing the maximum reaching distance of the tip of the hallux above the tape measure. The first leg to be tested, the first reaching direction, and the order of the consecutive reaching directions were randomised.

**Figure 1:**
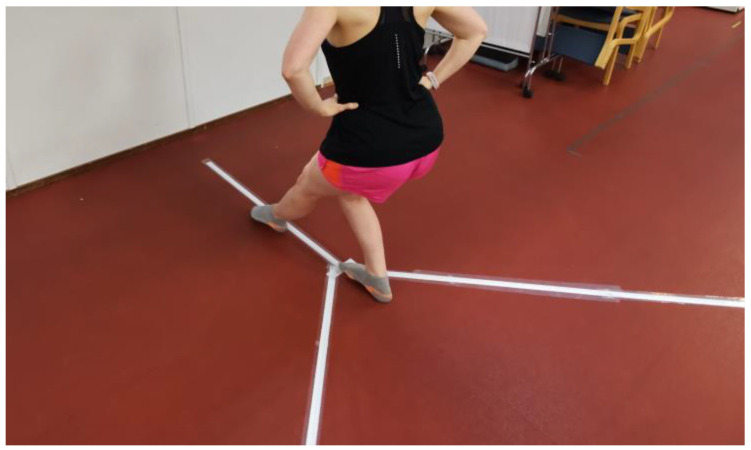
Measurement setup with the participant reaching in the anterior direction.

The participant stood without shoes on one leg at the centre of the Y figure as shown in Figure 1. Successful repetitions were determined by fulfilling the following conditions:

1. Participant’s standing leg was not allowed to rise above the floor, and the heel had to stay on the floor;
2. Hands had to remain on hips;
3. The reaching leg was not allowed to touch the floor;
4. Returning to the starting position had to be performed in a controlled fashion.

Between repetitions, the participant’s leg was allowed to touch the floor next to the standing leg.

The participants were allowed to complete one practice repetition in each reaching direction. Participants then performed a target of five successful repetitions in each direction. If the last try was longer by at least 1 cm than the previous ones, the participant was allowed to perform new reaches until the result did not improve over the threshold or until they reached a maximum limit of 12 repetitions. The minimum number of successful repetitions was four to be included in the analysis. The next direction for the same leg was then performed after all repetitions of the previous direction were completed.

### 2.3. Statistical Methods

Means and standard deviations were calculated for descriptive characteristics of both baseline and retest measurements. A paired T test was used to determine whether the mean difference between the two sets of measurements regarding participant characteristics was zero. The study population comprised 16 participants, but the reliability analysis was performed by separating participants’ dominant and nondominant leg results. For this reason, the true sample size for the reliability analysis was 32 (*n* = 32). The reliability of the YBT was assessed by performing separate analyses for all of the reaching directions. The reaching result was normalised to the leg length by dividing the reaching distance by the length of the reaching leg and then multiplying the result by 100.

Two-way mixed-effects absolute agreement intraclass correlation coefficients (ICCs) with 95% confidence intervals were used for relative reliability analyses. The ICC analyses were separately carried out for different methods of score calculation using the ASIS–hallux measurement as the primary method of leg length normalisation. The analysis was then reperformed using ASIS–medial malleolus measurements for leg length normalisation. The ICC was separately calculated for scores acquired from the average of the first three repetitions, the average of the best three repetitions, and the best repetition.

Population standard deviation (SD) was calculated as square root of the variance (SD = √(σ^2^)). Standard error of measurement (SEM) was calculated by multiplying the standard deviation of baseline test results by the square root of 1 minus the ICC (SEM = SD × √(1-ICC)) [18]. The minimal detectable change (MDC) was also calculated (MDC = 1.96 × SEM × √2) [18]. Effect size was calculated using Cohen’s d_av_ (d_av_ = difference in means/pooled SD) [19]. To assess the number of successful repetitions needed to reach plateauing of results, the proportion of the average result in every subsequent repetition was compared with each direction’s maximum result.

Statistical analyses were conducted with IBM SPSS Statistics 27 (International Business Machines Corporation, Armonk, NY, USA) except for the SEM and MDC values, which were calculated using Microsoft Excel version 16.53 (Microsoft 2021, Microsoft Corporation, Redmond, WA, USA).

## 3. Results

The study included 12 female, and 4 male participants aged 29 to 51 years. The mean age, height, body mass, BMI, and leg length measurements gathered at both the baseline test and retest are shown in Table 1. No statistically significant differences were found between test and retest measurements for these descriptive characteristics.

The intrarater reliability of the YBT was not greatly affected by the method of score calculation. (The ICC ranges were 0.818–0.864 for the first three repetitions for different reaching directions vs. 0.828–0.906 for the three best repetitions vs. 0.829–0.895 for the best repetition.) Although the mean test score slightly differed when comparing the different methods, mostly because of learning effects, it remained within the standard deviation ranges. The intrarater reliability of the test was not affected by the leg length measurement method. (The ICC ranges for all directions and score calculation methods were 0.821–0.906 for the ASIS–hallux measurement vs. 0.818–0.901 for the ASIS–medial malleolus measurement.) Although the reliability was not affected, the mean score of the test greatly differed between leg length measurement methods. For all these separate analyses, the SEM percentages ranged from 3.28 to 5.70, and the MDC scores ranged from 5.2 to 15.6, as shown in Table 2.

**Figure 2:**
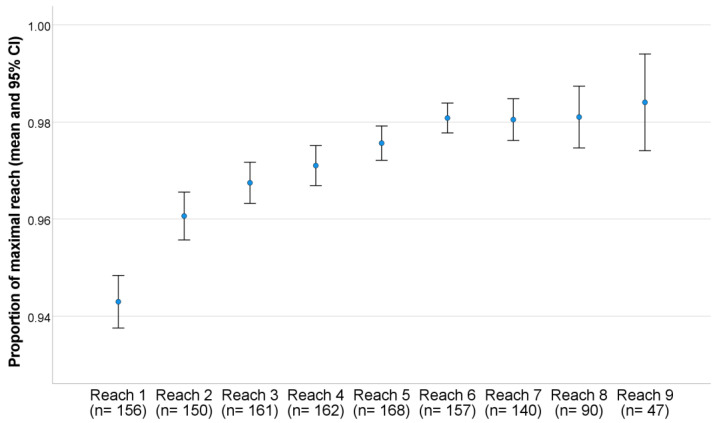
Plateauing of the results was reached after the sixth successful repetition.

In the analysis of potential learning effects, it was found that, on average, the test results plateaued after the sixth successful repetition per direction, making further repetitions redundant in terms of reliability. The number of repetitions shown in Figure 2 was limited to nine because there were only a few cases in which the participant completed more than nine successful reaches, which greatly affected the confidence interval shown.

## 4. Discussion

In this study, the intrarater reliability of YBT was found to range from good to excellent as measured by the ICC and confidence intervals. The reliability was not majorly affected by the method of score calculation or leg length measurement. As such, it is suggested that anterior superior iliac spine–medial malleolus measurement length is used for leg length normalisation, as it is the method proposed in the original YBT protocol [13]. It is also suggested that the average of the best three repetitions be used to mitigate possible outliers and account for the learning effects that were seen in this study. On a separate analysis of learning effects, a plateau of results was found after the sixth measurement, suggesting that further measurements would be futile.

The results of this study strengthen the position taken in earlier research that YBT is a reliable way to assess dynamic balance [15]. This further analysis performed on the different methodologies regarding ways to measure leg length for normalisation, number of repetitions, and score calculation paves the way for more consistency in using the YBT in both future research and clinical assessment.

Based on previous studies with differing protocols, an earlier review on reliability suggested using protocols with at least four practice repetitions followed by three collection repetitions [15]. The findings of the present study support this recommendation by providing data on the plateauing of the results at the sixth measurement, bringing the total to seven repetitions when the practice repetition is included. In studies conducted on YBT’s predecessor, SEBT, it was identified that plateauing of results seems to happen after the sixth measurement [20], which further strengthens these findings.

Future studies on injury risk as measured by the YBT should strive to use generally agreed-upon protocols. While this study provides suggestions to reach this goal, similar studies focused on different populations would complement these findings. While it seems that most parts of the YBT methodology have little impact on reliability, it is suggested that researchers and clinicians adopt a standardised number of repetitions and normalisation of results by leg length as a basis for more generalisable results.

### 4.1. Practical Applications

Based on these results, three recommendations can be made:

1. The landmarks used for measuring leg length seem to have a minimal effect on reliability. Due to this, it is recommended to use the ASIS–medial malleolus because it is the method proposed in the original YBT protocol [13];
2. At least 7 successful repetitions, including possible practice repetitions, should be conducted to reach the plateau of results, with a suggested cap of 12 repetitions;
3. As there were no clear differences in reliability between different methods of score calculation, it is recommended to use the average of the best three repetitions to try to mitigate any possible outlier results and to account for the learning effects seen in this study.

### 4.2. Study Limitations

The limitations of this study and recommendations come from having a somewhat homogenous group of 18- to 55-year-old participants with a similar novice recreational background in endurance running. Consequently, the results may not be fully applicable to populations comprising individuals under the age of 18 years, elderly people, or those with sedentary backgrounds. In addition, the sample size used was not calculated but was instead determined based on earlier studies showing sufficient statistical power with a similar sample size [13,20]. The second limitation relates to recommendations 1 and 3, where the reliability was analysed using the original study protocol regarding number of repetitions instead of the recommendation made after analysing the plateauing of the results. The third limitation of this study comes from two retests that were rated by a different pairing of raters. The primary rater stayed the same but, because of scheduling conflicts, the assisting rater had to be replaced by a similarly experienced rater. The effects of this limitation were tested by excluding the two participants’ results in question, and no relevant changes in ICC analysis were found except for increased confidence intervals because of having fewer participants.

## 5. Conclusions

YBT seems to have good intrarater reliability regardless of the methodology used. However, for results to be comparable between patients and clinicians, a consistent methodology regarding number of repetitions, method of score calculation, and leg length normalisation is needed.

## Figures and Tables

**Table 1:** Study participant characteristics.

Participants	Age (Years ± SD)	Height (cm ± SD)	Body Mass (kg ± SD)	BMI (kg/m^2^ ± SD)	A-H DOM (cm ± SD)	A-M DOM (cm ± SD)	A-H NDOM (cm ± SD)	A-M NDOM (cm ± SD)
								
Baseline test (*N* = 16)	39.1 ± 6.8	168.7 ± 6.2	74.4 ± 13.0	26.1 ± 3.9	105.5 ± 3.7	87.9 ± 3.3	105.4 ± 3.8	87.7 ± 3.4
Female (*n* = 12)	37.9 ± 6.9	166.9 ± 5.7	70.3 ± 8.0	25.3 ± 3.3	104.9 ± 3.7	87.6 ± 3.3	104.8 ± 4.0	87.4 ± 3.4
Male (*n* = 4)	42.5 ± 5.7	173.9 ± 4.4	86.5 ± 18.7	28.5 ± 5.1	107.4 ± 3.2	89.0 ± 3.8	107.1 ± 2.9	88.9 ± 3.7
								
Retest (*N* = 16)	39.1 ± 6.8	168.8 ± 6.2	74.1 ± 12.7	26.0 ± 3.8	105.6 ± 3.8	87.8 ± 3.3	105.6 ± 3.7	87.7 ± 3.2
Female (*n* = 12)	37.9 ± 6.9	167.0 ± 5.8	70.1 ± 7.8	25.2 ± 3.2	105.0 ± 4.0	87.4 ± 3.3	105.0 ± 3.8	87.3 ± 3.3
Male (*n* = 4)	42.5 ± 5.7	174.1 ± 4.4	86.2 ± 17.9	28.3 ± 4.9	107.6 ± 2.8	89.0 ± 3.2	107.3 ± 2.8	89.0 ± 3.0
								

BMI, body mass index; A-H, anterior superior iliac spine–hallux; A-M, anterior superior iliac spine–medial malleolus; DOM, dominant leg length; NDOM, nondominant leg length.

**Table 2:** Mean calculated scores for reaching directions, effect sizes, ICC values, SEM percentages, and MDC scores.

Reaching Direction	Baseline Test Mean ± SD (*n* = 32 *)	Retest Mean ± SD (*n* = 32 *)	Effect Size Cohen’s d_av_	ICC (95% CI)	SEM%	MDC
First three repetitions (A–H)						
Anterior	56.2 ± 4.1	54.7 ± 4.8	0.34	0.862 (0.659, 0.938)	4.26	6.6
Posteromedial	81.5 ± 8.1	82.5 ± 7.2	0.13	0.821 (0.636, 0.912)	5.59	12.7
Posterolateral	79.6 ± 7.5	78.9 ± 7.3	0.09	0.848 (0.689, 0.926)	5.15	11.3
First three repetitions (A–M)						
Anterior	67.5 ± 4.7	65.8 ± 5.6	0.33	0.864 (0.679, 0.938)	4.11	7.6
Posteromedial	97.8 ± 9.7	99.3 ± 8.9	0.16	0.818 (0.631, 0.911)	5.70	15.6
Posterolateral	95.5 ± 9.2	95.0 ± 9.1	0.05	0.838 (0.667, 0.921)	5.49	14.5
Three best repetitions (A–H)						
Anterior	57.6 ± 3.9	56.4 ± 4.6	0.28	0.906 (0.769, 0.958)	3.29	5.2
Posteromedial	84.5 ± 7.9	85.4 ± 6.7	0.12	0.828 (0.650, 0.916)	5.05	11.9
Posterolateral	82.3 ± 6.9	82.0 ± 7.0	0.04	0.894 (0.782, 0.948)	3.90	8.9
Three best repetitions (A–M)						
Anterior	69.1 ± 4.6	67.9 ± 5.4	0.24	0.901 (0.778, 0.954)	3.28	6.2
Posteromedial	101.5 ± 9.5	102.8 ± 8.3	0.15	0.828 (0.650, 0.916)	5.14	14.6
Posterolateral	98.9 ± 8.7	98.8 ± 8.8	0.01	0.891 (0.776, 0.947)	4.12	11.3
Best repetition (A–H)						
Anterior	58.2 ± 3.9	56.9 ± 4.6	0.31	0.894 (0.732, 0.953)	3.42	5.5
Posteromedial	85.3 ± 7.9	86.3 ± 6.7	0.14	0.829 (0.652, 0.916)	5.00	11.9
Posterolateral	83.4 ± 6.7	82.9 ± 6.9	0.07	0.895 (0.785, 0.949)	3.75	8.6
Best repetition (A–M)						
Anterior	69.9 ± 4.5	68.5 ± 5.4	0.28	0.885 (0.739, 0.947)	3.46	6.7
Posteromedial	102.4 ± 9.6	103.8 ± 8.3	0.16	0.830 (0.655, 0.917)	5.11	14.6
Posterolateral	100.1 ± 8.5	99.8 ± 8.6	0.04	0.891 (0.776, 0.947)	3.99	11.1

A–H, anterior superior iliac spine–hallux; A–M, anterior superior iliac spine–medial malleolus; SD, standard deviation; ICC, intraclass correlation coefficient; CI, confidence interval; SEM%, standard error of measurement percentage; MDC, minimal detectable change. * Both legs results included in the calculations.

## Data Availability

The data presented in this study are available on request from the corresponding author. The data are not publicly available due to privacy.
